# Is Micronucleus Assay Suitable for Cytogenetic Biomonitoring the Different Ways to Smoke?

**DOI:** 10.30699/ijp.2020.125206.2370

**Published:** 2020-07-31

**Authors:** Ingra Tais Malacarne, Daniel Vitor de Souza, Daniel A Ribeiro

**Affiliations:** 1 *Department of Biosciences, Federal University of São Paulo, UNIFESP, Santos, SP, Brazil*

Dear Editor,

We read the manuscript of DehghanNezhad *et al. *(1) recently published in the Iranian Journal of Pathology titled “Micronucleus Assay of Buccal Mucosa Cells in Waterpipe (Hookah) Smokers: A Cytologic Study.” with much interest. In this article, the authors were able to detect high frequencies of micronucleus in buccal mucosa cells of waterpipe smokers when compared to non-smokers. However, it is important to properly assess the scientific approach for a correct understanding of the paper. 

First, it is important to stress that some criteria were established for the correct identification of micronucleus in buccal mucosa cells. In Material and Methods part, the authors stated that “The structures within cytoplasm with similar staining of nucleus measuring between 1/5 to1/3 size of nucleus was considered as micronucleus”. However, Figure 1 does not fulfill these criteria, because micronuclei are not similar to that staining of nucleus and they are associated with distinct focus plane when compared to main nucleus. Certainly, Figure 1 was not stained with Feulgen-Fast Green since the cytoplasm of buccal cells is violet in color and not green. 

Another question is concerned to the total number of cells investigated. According to the Micronucleus Assay Expert Group, it is very important to evaluate a minimum of 2000 oral cells per volunteer (2). In this study, a total of 1000 cells were evaluated only by the authors. The approach is essential because the presence of micronucleus is very infrequent in the oral mucosa cells. 

In the Results, the mean data >1.0 from the micronucleus frequency in non-smokers was presented. Are the data presented as percentage in Table 1? This needs further clarification because it is assumed that the spontaneous micronucleus frequency in exfoliated buccal cells from health subjects is ranging from 0.7 to 0.8% (3). 

We believe that these comments can be useful for better understanding the relevant study on cytogenetic biomonitoring on buccal mucosa cells of waterpipe smokers.
